# Failure of reversion of neuromuscular block with sugammadex in patient with myasthenia gravis: case report and brief review of literature

**DOI:** 10.1186/s12871-019-0829-0

**Published:** 2019-08-17

**Authors:** Hermann dos Santos Fernandes, Jorge Luiz Saraiva Ximenes, Daniel Ibanhes Nunes, Hazem Adel Ashmawi, Joaquim Edson Vieira

**Affiliations:** 1 Anesthesia Division, Clinics Hospital of University of São Paulo Medical School, Av. Dr. Enéas de Carvalho Aguiar n° 155, 8° Andar, São Paulo, 05403000 São Paulo Brazil; 2 Rua Cardeal Arcoverde, 388. Apto 111, São Paulo, 05408000 São Paulo Brazil

**Keywords:** Myasthenia gravis, Neuromuscular blockade reversal, Sugammadex

## Abstract

**Background:**

Myasthenia gravis (MG) is a challenge for anesthesia management. This report shows that the use of rocuronium-sugammadex is not free from flaws and highlights the importance of cholinesterase inhibitors management and neuromuscular block monitoring in the perioperative period of myasthenic patients.

**Case presentation:**

Myasthenic female patient submitted to general balanced anesthesia using 25 mg of rocuronium. Under train-of-four (TOF) monitoring, repeated doses of sugammadex was used in a total of 800 mg without recovery of neuromuscular blockade, but TOF ratio (TOFR) was stabilized at 60%. Neostigmine administration led to the improvement of TOFR.

**Conclusions:**

Although the use of rocuronium-sugammadex seems safe, we should consider their unpredictability in myasthenic patients. This report supports the monitoring of neuromuscular blockade as mandatory in every patient, especially the myasthenic ones.

## Background

Myasthenia Gravis (MG) is an autoimmune disease that affects the neuromuscular junction and neuromuscular transmission, therefore it causes muscle weakness. The most common form involves antibodies against the nicotinic acetylcholine receptor (AchR), reaching up to 80% of the cases. The phenotype can also vary, with several muscle groups affected in different ways. The most commonly affected are the eyes muscles. The most serious manifestations are the myasthenic crisis (MC) and the cholinergic crisis [1].

MG patients are a challenge for anesthesiologists in several aspects. Antibiotics, sedatives, inhalational anesthetics and surgical stress can trigger its symptoms [1]. In this scenario, neuromuscular blocking agent (NMB) use increases the risk of residual paralysis. Succinylcholine is not recommended for myasthenia as it has a slower onset of action and a delayed recovery. The myasthenic patient has greater sensitivity to nondepolarizing NMB due to the reduced number of functional AChR [1]. Sugammadex may be a safe option in the reversal of neuromuscular blockade by rocuronium. This duet may be considered the first choice when neuromuscular block in MG patients is needed [2–7]. However, there are some cases in the literature that report failures with these drugs in myasthenic patients [8] as well as in patients without myasthenia [9]. The purpose of this case report is to highlight the importance of cholinesterase inhibitors management and neuromuscular block monitoring in the perioperative period of myasthenic patients, even with the use of rocuronium-sugammadex. Written informed consent was obtained from the patient.

## Case presentation

MG female patient, 27 years old, 110 kg, 172 cm, BMI 37.18 kg/m^2^, in use of azathioprine (150 mg qDay) and pyridostigmine (240 mg qDay), submitted to videolaparoscopic cholecystectomy. On the days before the surgery, her disease was stable, under pharmacological treatment, with no symptoms. No plasmapheresis was performed. At the morning of the day of the surgery, she received pyridostigmine 240 mg. Orotracheal intubation was performed by fiberoscopy, under topical anesthesia, as the patient had a closed previous tracheostomy, followed by venous induction after intratracheal cannula position confirmation. For neuromuscular block monitoring, an acceleromyography method device was used (TOF Watch®). Before the injection of rocuronium (20 mg – 01xED_95_ for ideal body weight), this device was calibrated, and the train-of-four ratio (TOF) ratio was 100%. Anesthesia was maintained with sevoflurane. The timeline of events during anesthesia is illustrated in Table 1. The patient was maintained under temperature control and monitoring. Warm air blanket device and pharyngeal thermometer were used. She had normal core temperature at all times (36–36.8 °C). The surgery had no intercurrences. She kept hemodynamic stability during all time of surgery. At the end of the surgery, the neuromuscular monitor showed one response to four stimuli. A first bolus dose of sugammadex 200 mg (equivalent to approximately 2 mg/kg, for body weight) was used at 3:50 PM. At 4:15 PM the TOF counting presented four responses and TOF ratio (TOFR) was 45%. A second bolus of 200 mg of sugammadex did not change the TOFR results. At 4:25 PM, another 200 mg was administered, followed by a slight improvement in neuromuscular monitor (TOFR of 50%). Extubation was performed on her awakening at 4:35 PM, as she was in adequate spontaneous breathing with minimal support by mechanical ventilator. She complained of respiratory discomfort, and 200 mg of sugammadex were injected at 4:40 PM without clinical improvement and no changes on neuromuscular monitor (TOFR of 60%). At this point, it was decided to administer neostigmine 2 mg and atropine 0,5 mg, at 4:50 PM, which resulted in a progressive improvement of respiratory pattern. At 5:00 PM, neuromuscular monitor showed TOFR of 100%. The patient was then maintained under supplemental O2 5L/min by facial mask and then referred to the ICU with no adverse events until final discharge to the ward.

**Table 1:** Summary and timing of perioperative events

Time	1:50 PM	2:00 PM	2:05 PM	3:00 PM	3:50 PM	4:15 PM	4:25 PM	4:35 PM	4:40 PM	4:50 PM	5:00 PM
Event	Awake intubation	Post intubation	Beginning of surgery	Intraoperative period	End of surgery	Espontaneous breathing	Inhaled agent turned off	Awaking and extubation	Respiratory discomfort	Respiratory discomfort	No respiratory discomfort
TOF Count (N responses) or TOF Ratio (%)	100%	–	0 response	3 responses	1 response	45%	50%	60%	60%	60%	100%
Propofol (mg)	–	100	–	–	–	–	–	–	–	–	–
Ketamine (mg)	–	–	50	–	–	–	–	–	–	–	–
Fentanyl (mcg)	250	250	150	100	–	–	–	–	–	–	–
Rocuronium (mg)	–	20	–	5	–	–	–	–	–	–	–
Sevoflurane expiractory fraction (%)	–	1.6	1.6	1.6	1.6	1.6	Turned off	–	–	–	–
Sugammadex (mg)	–	–	–	–	200	200	200	–	200	–	–
Neostigmine (mg)	–	–	–	–	–	–	–	–	–	2	–

## Discussion and conclusions

MG has long been a challenge for anesthesiologists. Plasmapheresis or administration of intravenous immunoglobulins prior to surgery have already been recommended for these patients [1]. Currently, these practices are reserved for patients with poor control of symptoms requiring surgery. In elective cases, it is better to perform surgery at best moment of disease control, with lower doses of symptomatic and immunosuppressive medications. Pre-anesthetic medications (benzodiazepines and other sedatives) should be avoided [7]. An alternative for the NMB is the use of inhaled anesthetics in high concentrations.

In many cases, however, if NMB is needed, empirical experience in several case reports indicates that reduction of 50% of the usual dose is recommended in these patients [1]. The choice of rocuronium-sugammadex is the preferred option in the current scenario, although the dose of sugammadex in myasthenia is not standardized yet. Case reports of success used doses of 2 to 4 mg/kg for moderate and intense blocks [2–7]. In our case, sugammadex was used in fractional doses of 200 mg (equivalent to 1,81 mg/kg) and guided by quantitative TOF monitoring, in a total of 800 mg (equivalent to 7,27 mg/kg) which should have been an effective dose.

Myasthenic crisis (MC) or worsening of myasthenia status may be another perioperative problem. Sepsis, use of corticosteroids, surgical stress, pregnancy, stop of immunosuppressive agents and use of drugs that interfere with neuromuscular junction can increase muscle weakness. In this case, in addition to surgical stress and NMB, sevoflurane was also used. These factors may be causes of decreased TOF measurements [1]. An important item to be considered is the treatment agent for MG, the cholinesterase inhibitors agents. It is possible that the delay in recovery of the TOFR resulted from falling of pyridostigmine blood levels. That might be the reason why the patient recovered so quickly after neostigmine administration [1].

To this date, no randomized studies have been conducted with sugammadex in the specific group of myasthenic patients. The majority of patients was reported on case series and case reports [1–5, 7]. Neuromuscular blockade with rocuronium and its reversal with sugammadex seems to be the best option, when NMB is needed [6, 7], but it does not dispense from the use of objective neuromuscular monitoring.

In general anesthesia, several conditions that may interfere in the recovery to the neuromuscular blockade should be considered, like the unpredictability of NMBs in patients with MG, the greater sensitivity to the non-depolarizing agents, the lack of standardized dose of NMBs in these patients and the precise magnitude of anesthetic drugs (hypnotic, opioids, volatile anesthetics) interference as well as other substances. The use of rocuronium-sugammadex may not be completely predictable without neuromuscular blockade monitoring, since patients with MG may manifest MC or even MG worsening in the perioperative period, regardless of NMB. The present report adds to the literature supporting to the use of neuromuscular blockade monitoring as mandatory for surgeries in patients with MG, especially in cases where NMB is required.

## Data Availability

Data are available on request due to privacy or other restrictions. The data that support the findings of this study are available on request from the corresponding author HSF. The data are not publicly available due to them containing information that could compromise research participant privacy/consent.

## Abbreviations

AchR: Acetylcholine receptor
MC: Myasthenic crisis
MG: Myasthenia gravis
NMB: Neuromuscular blocking agent
TOF: Train-of-four
TOFR: Train-of-four ratio
